# Single-Pixel Background Modeling Algorithm for Strong Sky Scenes Based on Local Region Spatial Bases

**DOI:** 10.3390/s23010522

**Published:** 2023-01-03

**Authors:** Biao Li, Zhiyong Xu, Jianlin Zhang, Quanyou Zhao

**Affiliations:** 1 School of Computer Sciences and Engineering, Guilin University of Aerospace Technology, Jinji Road, Guilin 541004, China; 2 Institute of Optics and Electronics, Chinese Academy of Sciences, Guangdian Avenue, Chengdu 610209, China; 3 University of Chinese Academy of Sciences, Yuquan Road, Beijing 100049, China

**Keywords:** background modeling, dim-small target, extremely low signal-to-noise ratio, strong sky scene, strong random vignetting

## Abstract

In the dim-small target detection field, background suppression is a key technique for stably extracting the target. In order to effectively suppress the background to enhance the target, this paper presents a novel background modeling algorithm, which constructs base functions for each pixel based on the local region background and models the background of each pixel, named single pixel background modeling (SPB). In SPB, the low-rank blocks of the local backgrounds are first obtained to construct the background base functions of the center pixel. Then, the background of the center pixel is optimally estimated by the background bases. Experiments demonstrate that in the case of extremely low signal-to-noise ratio (SNR < 1.5 dB) and complex motion state of targets, SPB can stably and effectively separate the target from the strongly undulant sky background. The difference image obtained via SPB background modeling has the characters: the non-target residual could be white noise, and the target is significantly enhanced. Compared with the other typical five algorithms, SPB remarkably outperforms other algorithms to detect the target of a low signal-to-noise ratio.

## 1. Introduction

It is difficult to stably detect a dim-small target whose size is no larger than 9 × 9 pixels and whose energy is weak. The dim-small target detecting ability is a critical performance index of a remote detection system. Therefore, dim-small target detection has always been a research hotspot in the field of target detection. In complex scenes, due to the interference of environmental clutters such as an undulant sky background, illumination, clouds, and man-made artifacts, the contrast between the target and the local background is extremely weak. Therefore, in complex scenes, in order to highlight and stably detect the target, it is necessary to suppress or eliminate the background.

In the field of dim-small target detection, there are two main kinds of classical background modeling algorithms, which are based on two different image models. The first kind considers the image as the composition of background, noise, and target. Typical algorithms of this kind include the Gaussian filtering algorithm [1,2], the top hat transformation algorithm [3], the median filtering algorithm [4,5], and so on. Their basic idea is that the local neighborhood pixels of the background are statistically correlated, and the background of the target could be properly fitted by its neighborhood background. This kind of algorithm directly estimates the background but may severely damage the target signal. The other kind of algorithm regards the image data as the composition of a low-rank background matrix and sparse matrix from the perspective of the mathematical matrix. Recently, many algorithms have been proposed to estimate the background based on the low-rank matrix, such as the robust PCA algorithm [6,7,8,9], IPI [10], and PSTNN [11]. They mine the matrix structure information of image data, so that the integrity of the target signal is better. From the perspective of the global characteristics of the algorithms, the common point of these two kinds of algorithms is that they use a global function with fixed parameters to estimate the background image without fully considering the change in local characteristics. The first kind of algorithm uses a global structural element (or sliding window) to estimate the background of the central pixel. The second kind of algorithm uses principal component analysis (PCA) to construct the global background estimation function and obtain the low-rank component of the image data matrix.

The high-altitude dim-small target detection scene under strong sunlight is called the strong sky scene. Unlike the ground scene and low altitude scene whose backgrounds are mainly composed of vegetation, water flow, artificial objects, clouds, and other natural environments, the background of the strong sky scene is mainly composed of noise and non-uniform vignetting. When the non-uniform vignetting is very strong, the SNR of the target will be severely weakened, and the target will almost be submerged in the strong vignetting background. In this case, the geometric feature and gray feature of the dim-small target almost completely disappear, which makes the algorithm of the first kind fail to achieve good results. Similarly, the strong vignetting makes the image excessively sparse, that is, the sparsity of the target and noise is greatly weakened, and the target becomes a part of the background. As a result, the second kind of algorithm cannot effectively separate the background and the target. Therefore, in order to solve the difficult problem that is how to effectively separate the dim-small target from the strong vignetting background, this paper presents a background modeling algorithm for a single pixel based on the local region background base (SPB).

The contribution of this paper is that it is a new pixel-level background estimation algorithm, which provides an effective background suppression method under the condition of extremely low SNR (SNR < 1.5 dB) for the strong vignetting sky scene. The characteristics of SPB are as follows:

- It is a background suppression algorithm based on a single frame. The background value of a single pixel is determined by the base of the neighborhood background space. Therefore, the background estimation of an image can be realized by using a single-frame image.
- Good robust performance can be achieved when the dim-small target with different characteristics. In the SPB algorithm, as the neighborhood background fluctuates, the weight coefficient of the background base for linear combination will be adjusted instead of adopting a global function with fixed parameters. Therefore, the algorithm can adapt to the fluctuation of the background and the change of SNR, velocity, and trajectory of the target.
- Residual noise has a high degree of white noise. Although the characteristics of each region of the image are different, the residual noise of the difference images has evident white noise characteristics, including equal distribution and spectrum fluctuation around the expected value.
- Good effect can be stably obtained for strong sky scenes under the condition of extremely low SNR (SNR < 1.5 dB). In the case of extremely low SNR, in terms of energy, there is almost no difference between the target gray value and that of the neighborhood; in terms of scale, there is almost no difference between the target scale and the noise scale. However, for these scenes, the SPB algorithm can well separate the background and the target, and the target can be significantly enhanced.

In the rest of this article, Section 2 mainly discusses the background modeling algorithms based on image composition and matrix mathematical composition analysis. In the Section 3, based on discussing the image characteristics of the strong sky scene, the model-building method and background estimation method of the SPB algorithm are given. Background suppression experiments and quality assessment were carried out in the Section 4. The Section 5 is a brief conclusion.

## 2. Related Works

For the dim-small target scene, the geometric and grayscale features of the target are not obvious, making the target almost indistinguishable from noise and background texture. Therefore, deep learning algorithms are generally not used to estimate the background. The focus of background estimation research is mainly on image structure and matrix mathematical component analysis.

From the perspective of image structure, the image is composed of a background, noise, and a target. Algorithms based on this cognitive perspective can fall into two main categories. The first category is to estimate the background according to the good continuity and correlation of the background region and then subtract the estimated background from the original video frame image to obtain the difference image that the target had enhanced. Furthermore, this kind of algorithm can be divided into three subclasses: the filtering algorithm, the statistical model algorithm, and the algorithm based on the classification problem. The filtering algorithm uses a structure element (sliding window) to obtain the neighborhood pixel grayscale information to estimate the background value of the center pixel. Common algorithms include anisotropy algorithm [12], top hat transformation algorithm [3], median filtering algorithm [4,5], mean filtering algorithm [1,4], bilateral filtering algorithm [13], two dimensional least mean square error algorithm [14], and so on. While suppressing the background, these algorithms also make the target information lose. For example, the target is smoothed, the target edge information is lost, etc. They are less adaptable when the background is complex, such as rich textures, diverse background types, and small areas. The statistical model algorithm establishes appropriate statistical models for various pixels to obtain better-estimated values for complex backgrounds. Gaussian mixture model algorithm [1,2] is the typical representative of this kind of algorithm. However, the real-time performance of this kind of algorithm is poor, mainly for the following reasons: The background model initialization requires sequence images, and the model learning needs to be redone when the background changes significantly; with the increase of the number of statistical models that are involved in mixing, the complexity of updating the model parameters will increase sharply. The background modeling algorithm based on the classification problem has a good real-time performance. The typical representative algorithms are VIBE [15,16] algorithm and its derivative algorithms. Such as PBAS [17], VIBE+ [18], etc. These algorithms only need a single frame of the image to build the sample set of each pixel, so the background model initialization can be done quickly. However, the most obvious disadvantages of this kind of algorithm are the presence of ghost areas, the absorption of stationary or slow-moving targets, shadow foreground, and incomplete moving targets. These shortcomings are closely related to the sample set update strategy for pixels. Different from the first category, which directly estimates the background, the second category is to make use of the singularity characteristics of the target to match, enhance and extract, so as to achieve the separation of target and background. There are two typical subclasses of this category: algorithms based on the human visual system and dictionary-matching lookup algorithms. The algorithm based on the human visual system obtains the target matching scale chart according to the salience of the contrast between the target and the neighborhood background [11]. Such as the difference between the Gaussian algorithm (DOG) [19], Laplacian of Gaussian algorithm (LOG) [20], RLCM [21], etc. The dictionary matching look-up algorithm is to establish the target dictionary according to the salient characteristics of the target, and the target can be confirmed when it matches the dictionary. Such as the works of the literature [22,23,24], etc. The most important deficiency of the second category algorithms is that the target must have good salience characteristics, that is, the target SNR must be high, otherwise the algorithm effect will be affected.

Analyzing the data components of the image matrix from the perspective of the mathematical matrix is a modeling method to explore the intrinsic law of image data. A typical representative of this approach is the low-rank background modeling algorithm based on principal component analysis (PCA) [7]. In order to improve the robustness of the principal component analysis algorithm and effectively apply it to high-dimensional data, Candès et al. [6] proposed a novel robust PCA algorithm (RPCA) and successfully applied it to signal processing and computer vision. This work has made PCA - based low-rank background modeling problems a hot topic in the recent decade. According to different applications, many algorithms have been proposed. Typically, a real data sequence will last a long time and may change slowly. In this case, the robust subspace tracing (RST) [8,9] model was proposed to reduce the computational complexity and achieve continuous processing power. The RST model assumes that long data sequences reside in low-dimensional subspaces. This model is beneficial to solving the problem of insufficient memory space and realizing continuous real-time processing. The previous algorithms obtain the observation data matrix by converting the image into the column or row vector of the matrix, which will destroy the original structural characteristics of the image. Different from the algorithms that were mentioned above, in order to maintain the structure of the image and make good use of it, an algorithm based on tensor (TRPCA) [25] to obtain observation matrix data was proposed. The aforementioned robust PCA algorithms, that is, RPCA, RST, and TRPCA use sequence images to form data matrix in background modeling applications, that is, background initialization needs to be realized by using sequential frame images. When the background changes fast or the target moves rapidly, the effect of the background estimation will be affected. Therefore, in order to adapt to the rapid change of background or target, the robust PCA background modeling algorithm for a single frame image based on patch-image has had more attention in the recent years. Such as IPI [10,26,27], IPT [28], PSTNN [11], etc. These algorithms assume that the background of a single frame image is composed of the same low-rank subspace and the observation data matrix is obtained by region segmentation of a single frame image.

For dim-small target detection, the biggest challenge is carring out background modeling under the condition of extremely low SNR (SNR < 1.5 dB), so that the background and target can be effectively separated. The main reason for this is that in the case of extremely low SNR, the energy and geometric features of the target are not prominent so the target is almost completely integrated into the background and almost indistinguishable from the noise. Therefore, most of the current algorithms mainly focus on the input SNR ≥ 1.5 dB and rarely involve the scene with very low SNR. In order to effectively separate the background and target of a strong sky scene under very low SNR, the SPB algorithm is proposed in this paper. SPB takes advantage of the robust PCA algorithms’ feature that makes good use of the image structure. The method of estimating single-pixel background values based on background bases that were proposed by the SPB algorithm can adapt to scenes with fluctuating backgrounds and achieve a good target enhancement effect under the condition of extremely low SNR.

## 3. SPB Algorithm

This section will introduce the SPB Algorithm. Firstly, according to the characteristics of strong sky scene images, the main difficulties that affect dim-small target detection were analyzed. Then, the low-rank and sparse properties of strong sky scene images in global and local regions were discussed. A single-pixel background modeling model based on the background space base of the local region was presented. Finally, the optimal estimation of the single-pixel background value was discussed in detail.

### 3.1. Image Characteristic Analysis

**Figure 1:**
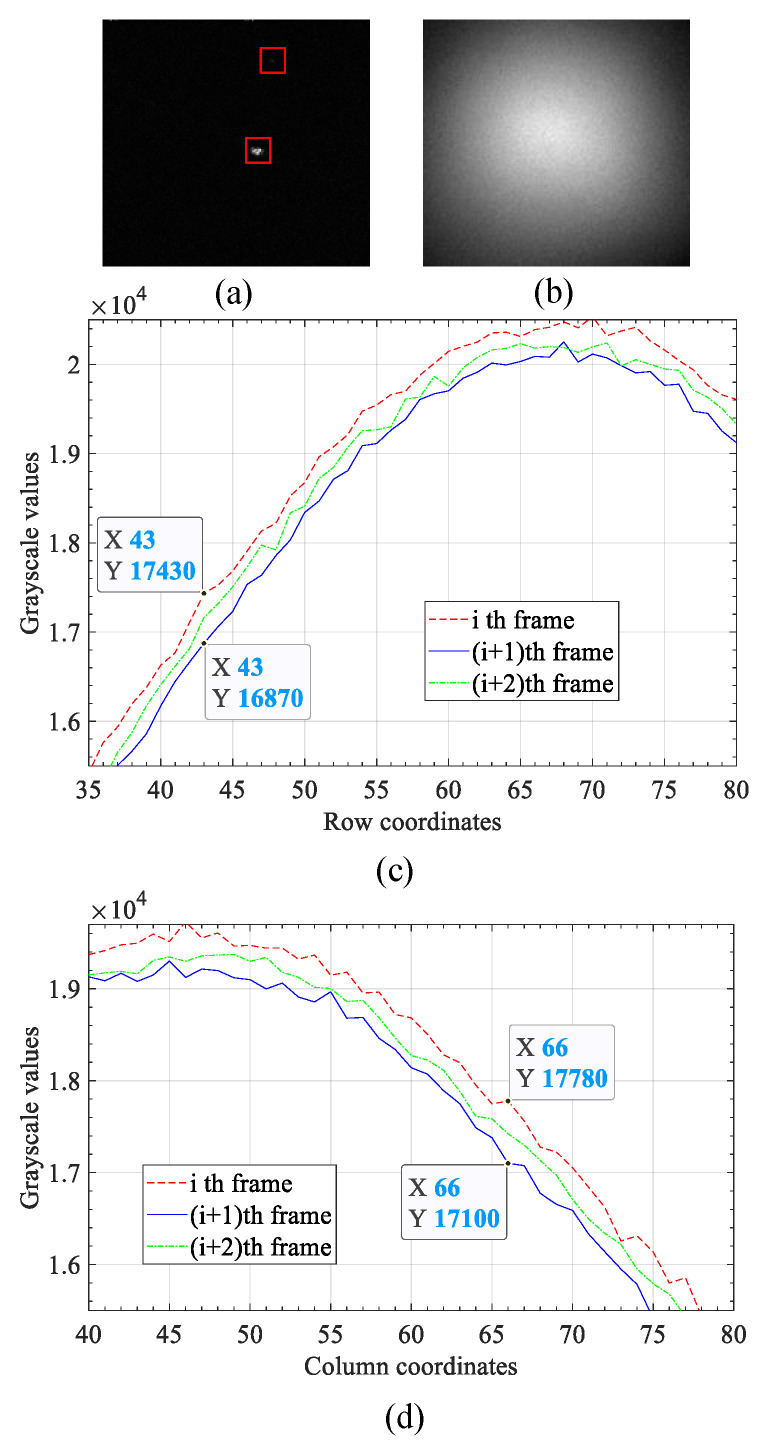
Analysis of vignetting characteristics. (**a**) There is no obvious vignetting on the image. (**b**) There is obvious vignetting on the image. (**c**) The curves of the summation of row pixel grayscale values of three random successive frames. (**d**) The curves of the summation of column pixel grayscale values of three random successive frames.

**Figure 2:**
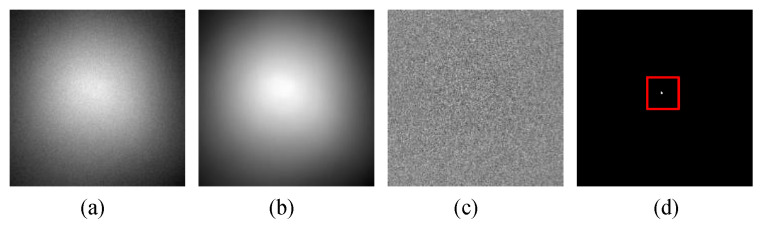
The sparse target cannot be separated effectively. (**a**) The original video frame image. (**b**) The low-rank background image. (**c**) The difference image. (**d**) The target image.

In the sky dim-small target detection, the main factors that affect the detection effect are the non-target photon noise and the dynamic random non-uniform strong vignetting caused by the system aperture effect. Figure 1a is the case where there is no non-uniformity phenomenon. Apart from noise, there is no obvious vignetting on the image, and the target is prominent. There is a serious non-uniformity phenomenon in Figure 1b. The target is drowned by the random strong vignetting, and the size and grayscale features of the target are completely impossible to be directly recognized by vision. Three successive frames are randomly selected from the sequence frame images, and then their row and column pixel grayscale values are summed respectively. The results of the summation of each row and column are shown as the curves in Figure 1c,d, respectively. As shown in the summation curves, the pixel values of the corresponding columns and rows between the previous and subsequent frames fluctuate significantly, which intuitively reflects the strong dynamic changes of the vignetting. From these analyses, it can be seen that there are three main difficulties affecting dim-small target detection. First, because of the wide distribution area and high gray value of the random non-uniform strong vignetting, the target and noise are submerged, making the sparsity component of the video frame image not obvious. In this case, if the algorithm of global robust PCA is used to carry out background estimation, the result is that the target is included in the low-rank background, that is, the difference image does not effectively contain the target. For example, Figure 2a is the original video frame image, on which there is a target as shown in Figure 2d. Figure 2b,c are the low-rank backgrounds and difference images that were obtained by the algorithm of global robust PCA, respectively. Obviously, due to the weak sparsity of the target in the original image, the target was integrated into the strong vignetting background, so that the algorithm of global robust PCA could not effectively separate the target from the background. Second, the distribution of vignetting has the characteristics of randomicity. The distribution of vignetting may be circular, elliptical, or other shapes depending on the working environment and the mode of movement of the imaging system. At the same time, the strength of each local area of vignetting is also random. The random distribution of vignetting will affect the adaptability of the background modeling algorithm to different scenes. Third, the random non-uniform strong vignetting is dynamic change. This dynamic change will weaken the correlation of the background image in the time domain and affect the effect of the background modeling algorithm based on the sequential frame image.

**Figure 3:**
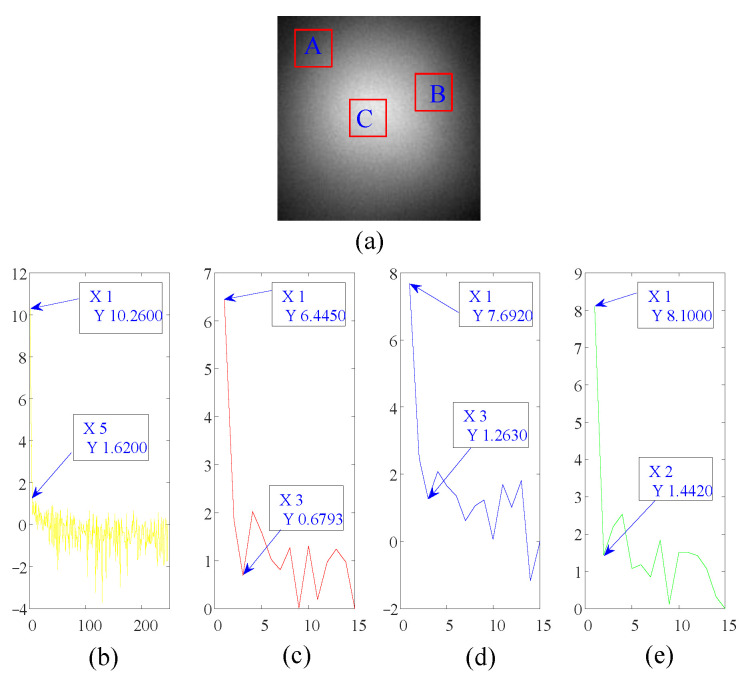
The global and local low-rank analysis of random non-uniform strong vignetting image. (**a**) Original video frame image. A, B, and C are three randomly selected local regions, and their scale is 15 × 15. (**b**) The singular value gradient logarithmic curve of the whole image. (**c**–**e**) are the singular value gradient logarithmic curves of the regions A, B, and C, respectively.

**Figure 4:**
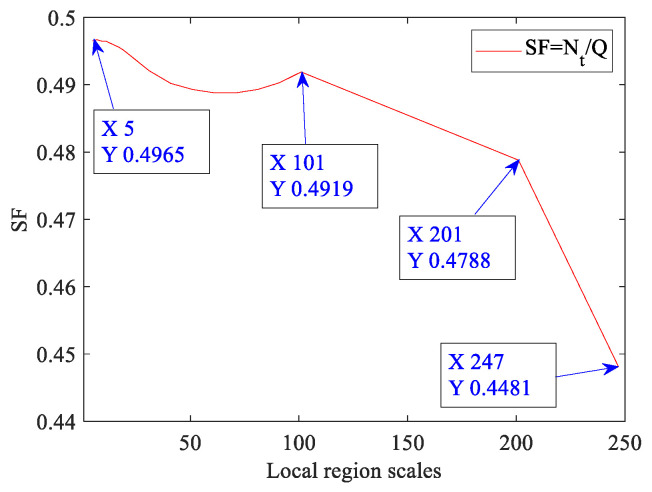
The sparse factor curve.

The rank of a matrix is equal to the number of non-zero singular values of the matrix, and the sparsity of a matrix can be measured by the sparse factor (SF). The definition of sparse factor is shown in Equation (1).

(1) $$\mathrm{SF}=\frac{N_{t}}{Q}$$

where *t* is the set of elements whose values are greater than the threshold value *T*, $t=\left\{x_{i}|x_{i}>T,\;x_{i}\in D\right\}$, $x_{i}$ is the *i*th element of ***D***. *T* takes the mean value of matrix ***D***. $N_{t}$ and *Q* are the total number of elements of *t* and ***D***, respectively. The low-rank and sparsity features of global (the whole image) and local regions are discussed below. Figure 3 is the global and local low-rank characteristic analysis diagram of random non-uniform strong vignetting images. In Figure 3a, A, B, and C are three randomly selected local regions. To enhance readability, the logarithm of the singular value gradient is taken to plot the curve. Figure 3 shows that the global singular value gradient becomes flat after the fifth singular value, and the local singular value gradient curve becomes flat after the second or third singular value. It shows that they all have good low-rank characteristics. Figure 4 is the sparse factor curve reflecting the global and local sparse characteristics. Table 1 shows the data of the sparse factors and their corresponding regional scale. The data in Figure 4 and Table 1 shows that as the local regional scale decreases, the sparse factor increases. This reflects that for strong vignetting images, the local region is more obvious sparsity than the global one, that is, the proportion of sparse elements in the local region is higher than that in the global one. Therefore, the analysis data of low-rank characteristics and sparse characteristics shows that the local region maintains appropriate sparsity, that is, avoids the occurrence of over-sparsity phenomenon, and still retains good low-rank characteristics.

The threshold value *T* of each local region takes the mean value of their grayscale.

### 3.2. SPB Background Model Modeling

#### 3.2.1. Model Analysis

**Figure 5:**
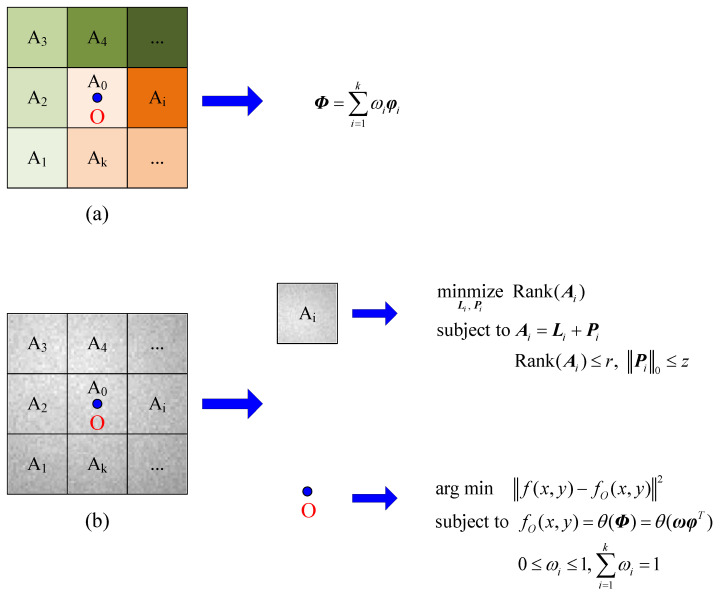
The schematic diagram of SPB algorithm. (**a**) The background model of region B in the presence of interference. (**b**) The schematic diagram of strong vignetting background modeling.

Figure 5 is the schematic diagram of the single-pixel background modeling algorithm (SPB). In practice, the background is always disturbed. Figure 5a is the background model of region B in the presence of interference. In order to make good use of the characteristic information of the background region to estimate the background value of the single pixel, region B is divided into several smaller local blocks. Thus, these local blocks have better consistent properties than region B. Assume that the background basis function of the *i*th local block $A_{i}$ is $\varphi_{i}$. As a result of the continuity of the background region, the basic function Φ of the real background of the local block where pixel O is located can be estimated by the background basis functions of all the local blocks, i.e.,

(2) $$\Phi =\sum_{i=1}^{k}\omega_{i}\varphi_{i}$$

$\omega_{i}$ is the weight coefficient, $0\leq \omega_{i}\leq 1$, $\sum_{i=1}^{k}\omega_{i}=1$, i=1, …, k.

Figure 5b is the schematic diagram of strong vignetting background modeling. The previous analysis of the data in Figure 3 and Figure 4 and Table 1 shows that the vignetting local block has a low rank and sparsity characteristics. Therefore, a low-rank background model can be established for the local block $A_{i}$, that is

$$\underset{L_{i},\;P_{i}}{\mathrm{minmize}}\;\;\mathrm{Rank}(A_{i})$$

$$\mathrm{subject}\;\mathrm{to}\;A_{i}=L_{i}+P_{i}$$

(3) $$\;\mathrm{Rank}(A_{i})\leq r,\;\;{\left\|P_{i}\right\|}_{0}\leq z$$

where, $L_{i}$ and $P_{i}$ are low-rank background matrix and sparse component matrix, respectively. ${\left\|\cdot \right\|}_{0}$ is the zero norm, and Rank(⋅) is the rank of a matrix. Both *r* and *z* are positive real numbers. The low-rank background $L_{i}$ can be obtained from Equation (3), and then the basis function $\varphi_{i}$ of $L_{i}$ can be found. Thus, as Equation (4), the optimal estimation model of the background value of the central pixel O can be established.

$$\arg\min\;\;{\left\|f(x,y)-f_{O}(x,y)\right\|}^{2}$$

$$\mathrm{subject}\;\mathrm{to}\;\;f_{O}(x,y)=\theta (\Phi )=\theta (\omega \varphi^{T})$$

(4) $$0\leq \omega_{i}\leq 1,\;\sum_{i=1}^{k}\omega_{i}=1$$

where, f(x,y) is the grayscale value of pixel O, and $f_{O}(x,y)$ is its optimal background value. θ(⋅) is a nonnegative function of Φ. $\omega =\left\{\omega_{i},\;i=1,\;\ldots \;,\;k\right\}$, $\varphi =\left\{\varphi_{i},\;i=1,\;\ldots \;,\;k\right\}$.

#### 3.2.2. Background Estimation

For the strong vignetting background, when the frame image has the over-sparsity phenomenon, the previous analysis shows that the appropriate sparse feature of the local region can be used to obtain the low-rank background. Then, according to the continuity of the local background, the background value of the central pixel is estimated by using the background space basis function of the local region.

For simplicity, $A_{i}$, $L_{i}$, and $P_{i}$ will be written in terms of ***A***, ***L***, and ***P*** for brevity when there is no ambiguity. Here, rewrite Equation (3) as follows

$$\underset{L,\;P}{\mathrm{minmize}}\;\;\mathrm{Rank}(A)$$

subject to A=L+P

(5) $$\;\mathrm{Rank}(A)\leq r,\;\;{\left\|P\right\|}_{0}\leq z$$

Equation (5) is a typical principal component analysis model. According to RPCA theory [6,7,8,29], it can be transformed into the principal component tracking model of the following equation for discussion

$$\mathrm{minimize}\;\;{\left\|L\right\|}_{*}+\lambda {\left\|P\right\|}_{1}$$

(6) subject to A=L+P

where, ${\left\|\cdot \right\|}_{*}$ is the nuclear norm, and ${\left\|\cdot \right\|}_{1}$ is the 1 norm. λ is the parameter that balances the terms ***L*** and ***P***, 0< λ < 1. ***A*** is the local block. ***L*** is the background matrix. ***P*** is the sparse matrix, which is composed of noise and potential target. According to the optimization theory [30,31,32], in order to improve the universality, the augmented Lagrange optimization algorithm is used to find the optimal solution of (6), i.e.,

(7) $$l(L,P,Y)={\left\|L\right\|}_{*}+\lambda {\left\|P\right\|}_{1}+\langle Y,A-L-P\rangle +\frac{\mu }{2}{\left\|A-L-P\right\|}_{F}^{2}$$

where ***Y*** is the Lagrange multiplier. μ is the penalty parameter, μ>0. By direct repeated iteration and update, the optimal solution of Equation (7) can be obtained, but the computing complexity is high. In order to reduce the complexity, the optimization method uses the soft-thresholding operator [30] and the singular value thresholding operator [33], i.e.,

(8) $$T_{t}[x]=\mathrm{sgn}(x)\max(\left|x\right|-t,0)$$

(9) $$H_{t}(X)=UT_{t}(\Lambda )V^{*}$$

where, x∈R, t>0. For two matrices, ***G*** and ***M***, with the same scale, a new matrix ***R*** is obtained through the soft threshold operation by traversing all the elements, i.e., $R=T_{G}[M]=T_{g_{ij}}[m_{ij}]$. Here, $g_{ij}$, $m_{ij}$ are the elements of the matrices ***G*** and ***M***, respectively. $X=U\Lambda V^{*}$ is singular value decomposition of matrix ***X***. Thus, the optimal solution of Equation (7) is obtained. That is

$$\begin{array}{l} \underset{L}{\arg\min}\;\;l(L,\;P,\;Y)=H_{\frac{1}{\mu }}(A-P+\mu^{-1}Y)\\ \underset{P}{\arg\min}\;\;l(L,\;P,\;Y)=T_{\frac{\lambda }{\mu }}(A-L+\mu^{-1}Y) \end{array}$$

(10) $$Y_{k+1}=Y_{k}+\mu (A-L_{k+1}-P_{k+1})$$

It is well known that the local regional background has strong consistency. Therefore, in order to estimate the background value of the center pixel of the region, the operations as follow are carried out: the eigenvector $\varepsilon_{i}$ of the $L_{i}$ local block background matrix composes the basis functions, then the background value $f_{O}(x,y)$ of the center pixel O can be modeled on these basis functions, i.e.,

(11) $$f_{O}(x,y)=\theta (\Phi )=\frac{1}{n}\sum_{j=1}^{n}\omega \varepsilon^{T}$$

Here, the nonnegative function θ(⋅) is the mean function. $\omega =\left\{\omega_{i},\;i=1,\;\ldots \;,\;k\right\}$ is the weighting coefficient, $0\leq \omega_{i}\leq 1$, $\sum_{i=1}^{k}\omega_{i}=1$. $\varepsilon =\left\{\varepsilon_{i},\;i=1,\;\ldots \;,\;k\right\}$ is the basis function vector. $\varepsilon_{i}$ is the eigenvector of the background matrix $L_{i}$. *n* is the number of all pixels in a local block.

Now, the specific expression of the SPB can be written as Formula (12)

$$\arg\min\;\;{\left\|f(x,y)-f_{O}(x,y)\right\|}^{2}$$

$$\mathrm{subject}\;\mathrm{to}\;\;f_{O}(x,y)=\frac{1}{n}\sum_{j=1}^{n}\omega \varepsilon^{T}$$

(12) $$0\leq \omega_{i}\leq 1,\;\sum_{i=1}^{k}\omega_{i}=1$$

For conciseness, in the case of without causing ambiguity, f(x,y) and $f_{O}(x,y)$ are abbreviated as f and $f_{O}$. Then Equation (12) can be written as following

$$\arg\min\;\;{\left\|f-f_{O}\right\|}^{2}$$

$$\mathrm{subject}\;\mathrm{to}\;\;f_{O}=\frac{1}{n}\sum_{j=1}^{n}\omega \varepsilon^{T}$$

(13) $$0\leq \omega_{i}\leq 1,\;\sum_{i=1}^{k}\omega_{i}=1$$

The Lagrange multiplier method [32] is used to find the optimal solution of Equation (13). First, Equation (13) is transformed into

(14) $$l(\omega ,\gamma )={\left\|f-\frac{1}{n}\sum_{j=1}^{n}\omega \varepsilon^{T}\right\|}^{2}+\gamma (\sum_{i=1}^{k}\omega_{i}-1)$$

Then, the derivative method is used to find the optimal solution of ω and γ

$$\underset{\omega_{i}}{\arg\min}\;\;l(\omega ,\gamma )=\frac{\partial l(\omega ,\gamma )}{\partial \omega_{i}}$$

(15) $$\underset{\gamma }{\arg\min}\;\;l(\omega ,\gamma )=\frac{\partial l(\omega ,\gamma )}{\partial \gamma }$$

γ is the Lagrange multiplier.

**Figure 6:**
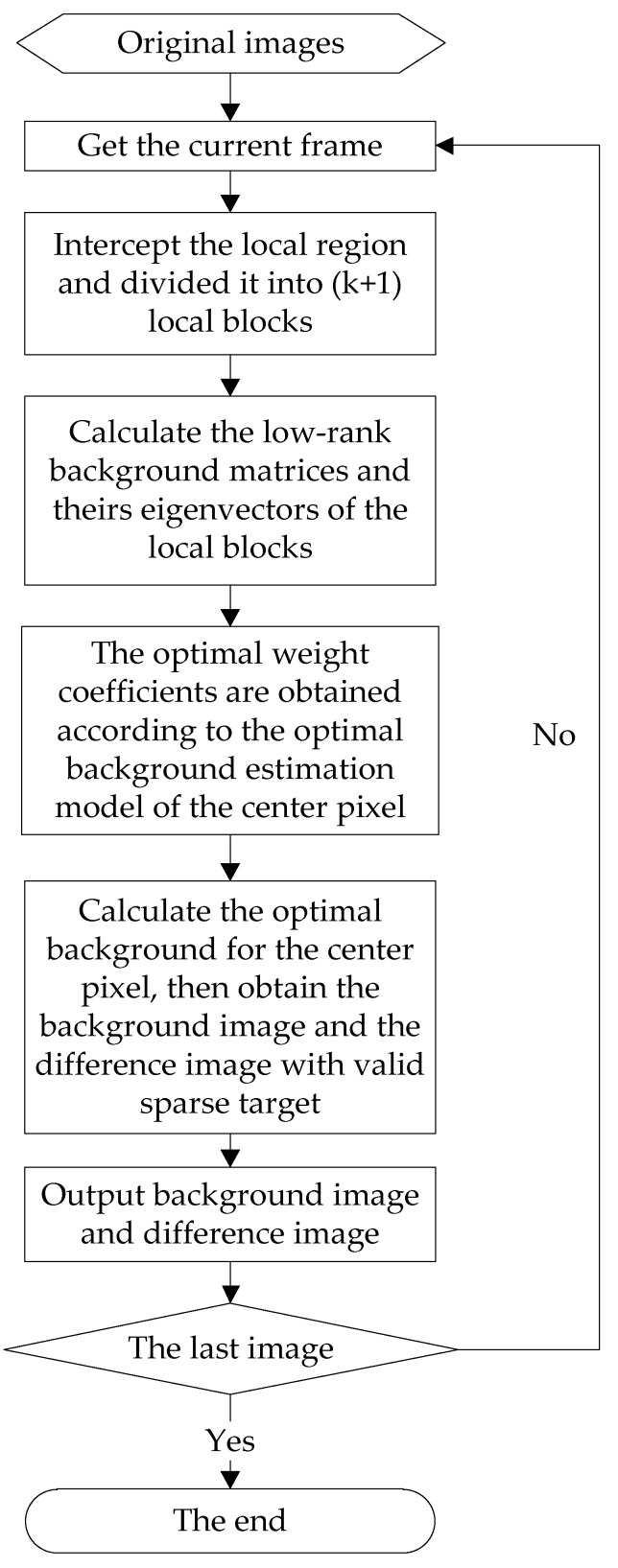
The flow diagram of the SPB methods.

In order to facilitate the understanding of the SPB algorithm, according to the above analysis, the pseudo-code and the flow diagram of the SPB algorithm are given out in Algorithm 1 and Figure 6.
**Algorithm 1** The pseudo code of SPB algorithm**Input:** Original video frame images $I\in R^{o\times p}$. **Output:** Background images $I_{L}$ and difference images $I_{P}$. **For**
*i*0 = 1:num//num is the total number of frames of the video.  1. Read in the *i0*th frame image ***I***, and get its size *m* and *n*.  2. Sliding the window **W** on the image ***I*** pixel by pixel to intercept the local region ***B***.  3. ***B*** is divided into (*k* + 1) local blocks $A_{i}$, i=0,1, … , k.  4. The low-rank background $L_{i}$ of local block $A_{i}$ is calculated by using the Equation (10).  5. Find the eigenvector $\varepsilon_{i}$ of the background matrix $L_{i}$.  6. The optimal weight coefficient $\omega_{i}$ is calculated according to the Equation (15).  7. The optimal background value $f_{O}$ of the central pixel O is estimated out by using the Equation (11).  8. Obtain the difference image: $I_{P}=I-I_{L}$. **End for**  9. Output $I_{L}$, $I_{P}$.

## 4. Experiments

In this part, experiments were carried out to verify the performance of SPB in suppressing the background and enhancing the target. Based on the experimental results, the robustness of the SPB algorithm is analyzed for the targets with different characteristics, the characteristics of the SPB residual noise are discussed, and a comparison with five other state-of-art algorithms is described. Furthermore, the experimental results are used to quantitatively analyze the operating range (OR), SNR gain (SNRG), and background suppress factor (BSF) of the SPB algorithm.

### 4.1. Experimental Environment

The experiments were carried out with OCTAVE on a computer running a 32-bit Windows 7 system, with a Core-i5 CPU, and 3 GB of RAM. For the strong sky scenes, in order to demonstrate the SPB algorithm performance of the background suppression and the dim-small target enhancement, three typical strong sky scenes with strong random vignetting are used for the experiments. The center area of the vignetting of scene A is large, scene B has random textures floor, and the vignetting of scene C changes significantly all the time. Five robust PCA algorithms: PCP [6], FPCP [34], OSTD [35], IPI [10], and PSTNN [11] were used as comparison algorithms. The experimental parameters of the comparison algorithms are shown in Table 2, where ***D*** is the data matrix or tensor. The evaluation indexes mainly include the Spectrum diagram, energy distribution diagram, SNR, SNRG, BSF, and operating range (OR). The definitions of SNR, SNRG [36], BSF, and OR are as follows

(16) $$\mathrm{SNR}=10\mathrm{lg}(\frac{\alpha_{t}-\alpha_{l}}{\delta_{l}})$$

(17) $$\mathrm{SNRG}=10\mathrm{lg}(\frac{{\mathrm{SNR}}_{\mathrm{out}}}{{\mathrm{SNR}}_{\mathrm{in}}})$$

(18) $$\mathrm{BSF}=\frac{\delta_{\mathrm{in}}}{\delta_{\mathrm{out}}}$$

(19) $$\mathrm{OR}\geq \min({\mathrm{SNR}}_{\mathrm{in}})$$

where, $\alpha_{t}$ and $\alpha_{l}$ are the grayscale mean values of the target and the target neighborhood region, respectively. $\delta_{l}$ is the grayscale standard deviation value of the target neighborhood region. ${\mathrm{SNR}}_{\mathrm{in}}$ and ${\mathrm{SNR}}_{\mathrm{out}}$ are the input and output SNR of the images before and after the background suppression. $\delta_{\mathrm{in}}$, $\delta_{\mathrm{out}}$ are the grayscale standard deviation values of the images before and after the background suppression. Here, the target neighborhood region scale is 3 times the target scale. The operating range (OR) is used to measure the input signal-to-noise ratio (SNR) range in which the algorithm can stably and effectively detect the dim-small targets. For dim-small target detection, generally, the algorithm can effectively detect the target with a high input SNR, and the detection effect of the algorithm is affected by a low input SNR. Therefore, the OR is determined by the minimum input SNR of the target that the algorithm can detect.

### 4.2. Quality Estimate

This section mainly discusses the performances of the background suppression and target enhancement of the SPB algorithm for the strong sky scenes from three aspects: the robustness of SPB, the suppression effect of SPB on the vignetting background, and the comparison of the experimental results with other five state-of-the-art methods.

#### 4.2.1. Robustness to the Targets with Different Characteristics

**Figure 7:**
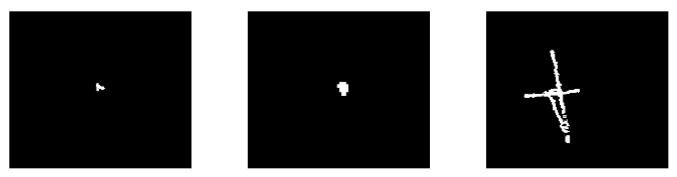
The target movement tracks of scenes A, B, and C.

For the strong sky scenes, the characteristics of dim-small targets mainly include the ratio of target signal energy to its neighborhood background energy (signal-to-noise ratio, SNR), target velocity, and target trajectory. The robustness of these characteristics is an important factor to measure the applicability of an algorithm to different strong sky scenes. The characteristics of the dim-small targets in these three scenes are listed in Table 3. The mean values of input SNR (${\mathrm{SNR}}_{\mathrm{in}}$) range from −0.2066 dB to 3.0237 dB. The ${\mathrm{SNR}}_{\mathrm{in}}$ values of a single frame image vary from −12.0820 dB to 11.1423 dB. The target speed ranges from 0 pixels per frame to 2 pixels per frame. The target in scene A first moves quickly up and then slowly to the lower right. In scene B, the target moves slowly all the time. In scene C, the target reciprocating moves quickly up and down, then left and right. In Figure 7, the target movement tracks of scenes A, B, and C are shown from left to right. The target trajectory in scene A is close to an inverted V. In scene B, the target swings near the original position. In scene C, the target reciprocating moves with a complex trajectory.

**Figure 8:**
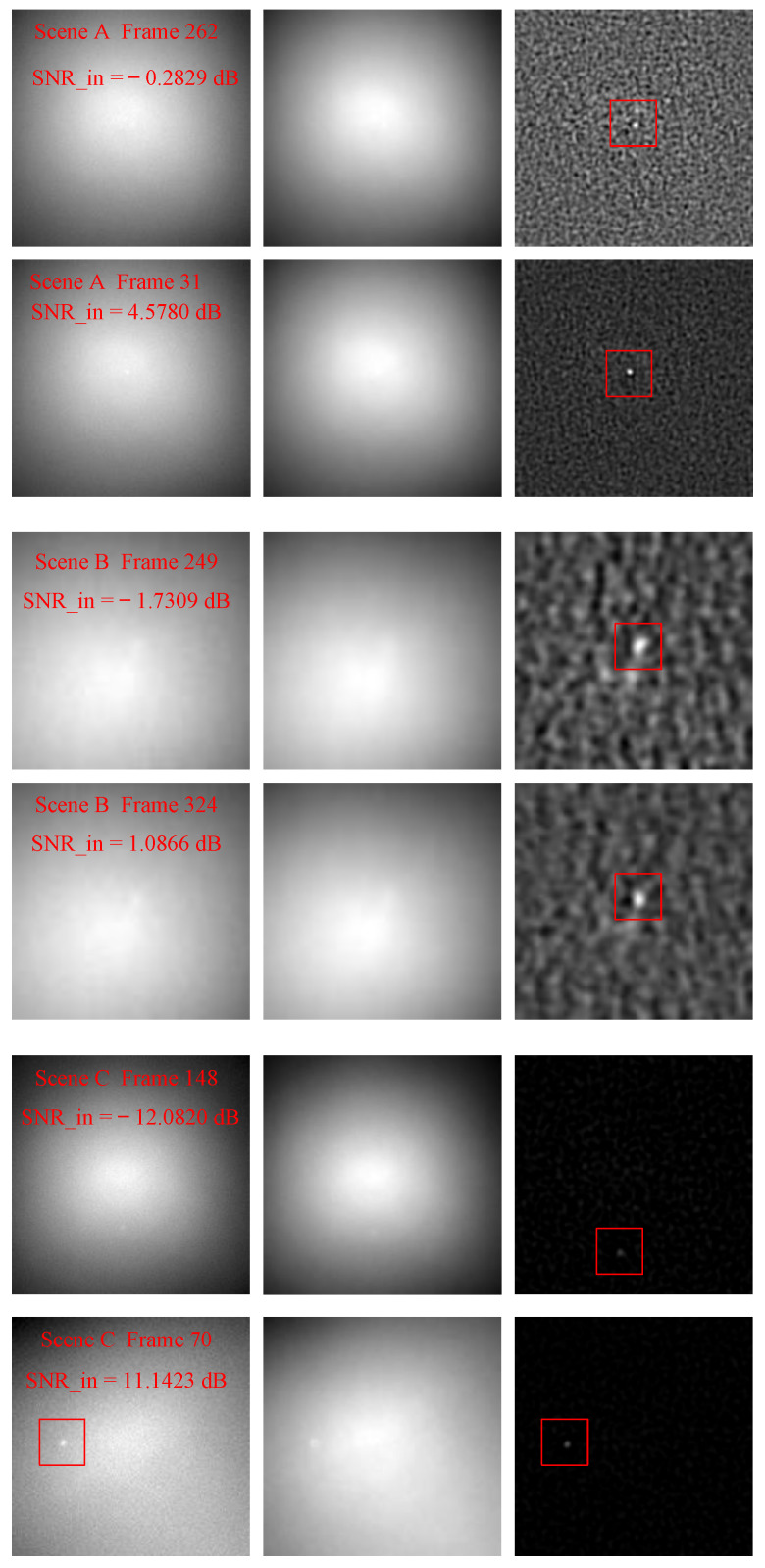
The background suppression effect of SPB when the target SNR is minimum and maximum of scenes A, B, and C.

**Figure 9:**
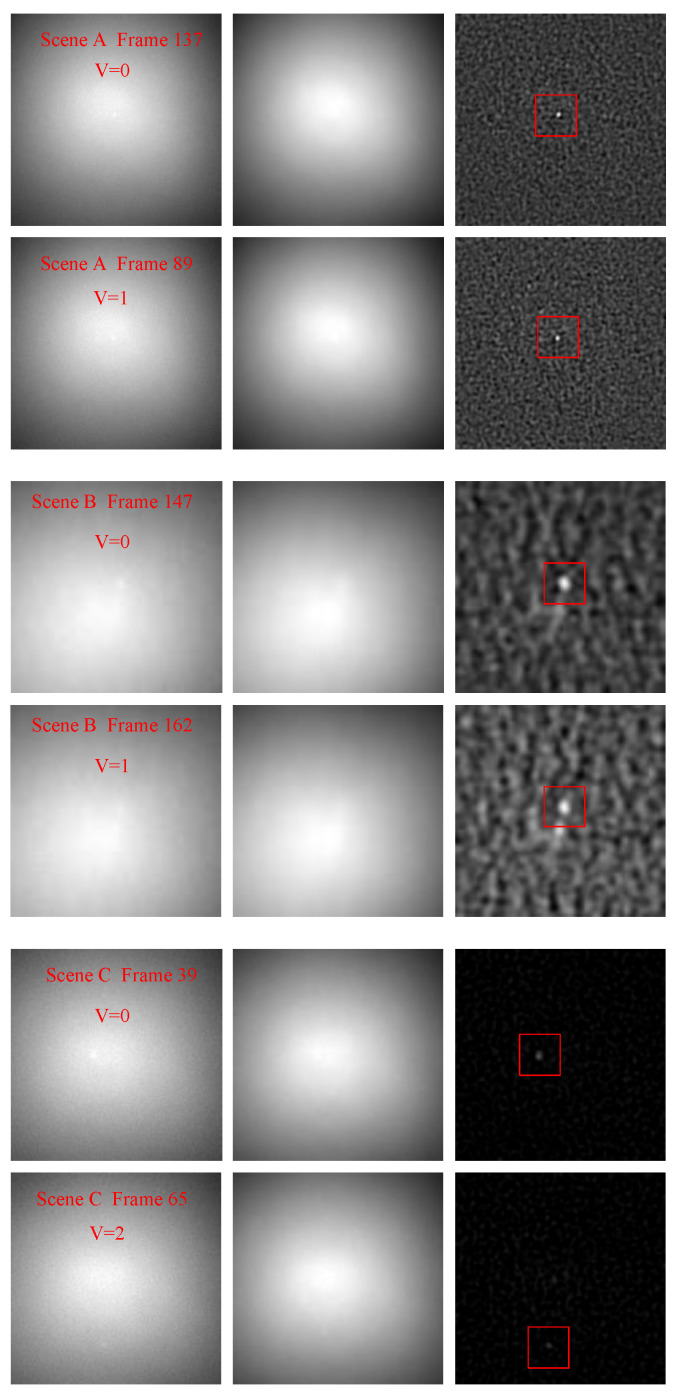
The background suppression effect of SPB when the target velocity is minimum and maximum of scenes A, B, and C.

**Figure 10:**
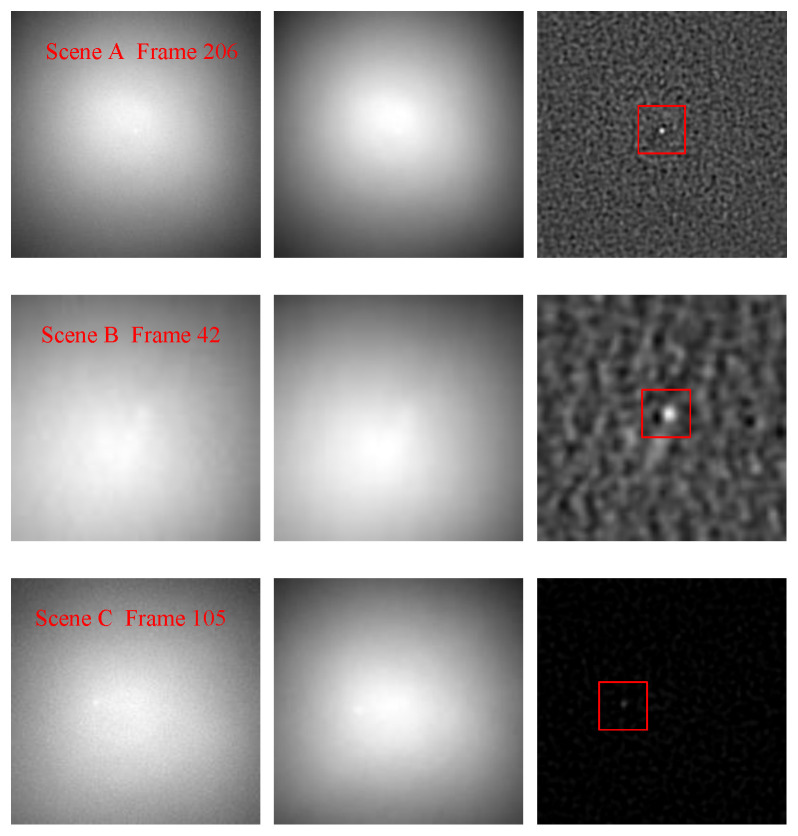
The background suppression effect of SPB under the different motion trajectories of the targets of scenes A, B, and C.

Figure 8 is the difference images when the target SNR in each scene is minimum and maximum, respectively. Figure 9 shows the difference images when the target velocity in each scene is minimum and maximum, respectively. For each scene, one frame was randomly selected when the target was moving on the trajectory, and their difference images are showed in the Figure 10. As demonstrated in Figure 8, Figure 9 and Figure 10, the SPB algorithm can effectively suppress the background and enhance the target when the target is in an extremely low SNR, moving at different speeds and different trajectories, that is, the SPB algorithm is robust to targets with different characteristics, and is adaptive to different strong sky scenes.

#### 4.2.2. Analysis of Residual Noise Characteristics

**Figure 11:**
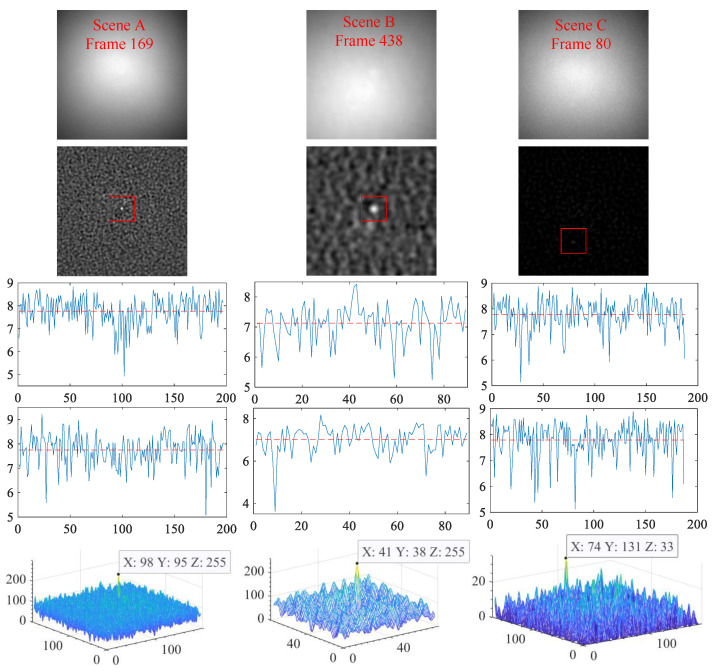
Whitening effect of the residual noise.

For strong sky scenes, the purpose of the SPB algorithm is to suppress the background and whiten the residual noise of the difference image, that is, the statistical characteristics of the residual noise conform to the characteristics of white noise, and the target is significantly enhanced. One frame image was randomly selected from each scene, and the spectrum characteristics and energy distribution of their difference images obtained by SPB are shown in Figure 11. In this figure, the method to obtain the spectrum of the difference images is to randomly select a row and a column, and then draw the spectrum curves of this row and column, respectively. As can be seen from Figure 11, the spectrum of the difference images of these three scenes fluctuates around the mean line, which is approximate to the spectrum of white noise. The residual noise in the difference images is evenly distributed, and the target energy is significantly stronger than that of the noise. Therefore, it can be seen that the SPB algorithm whitens the residual noise, and significantly enhances the target.

The first row is the original video frame images. The difference images are in the second row. The third and fourth rows are the spectrum images of a row and a column that were randomly selected from the difference images. The fifth row is the three-dimensional energy distribution diagrams of the difference images.

#### 4.2.3. Visual Comparison with Other Algorithms

**Figure 12:**
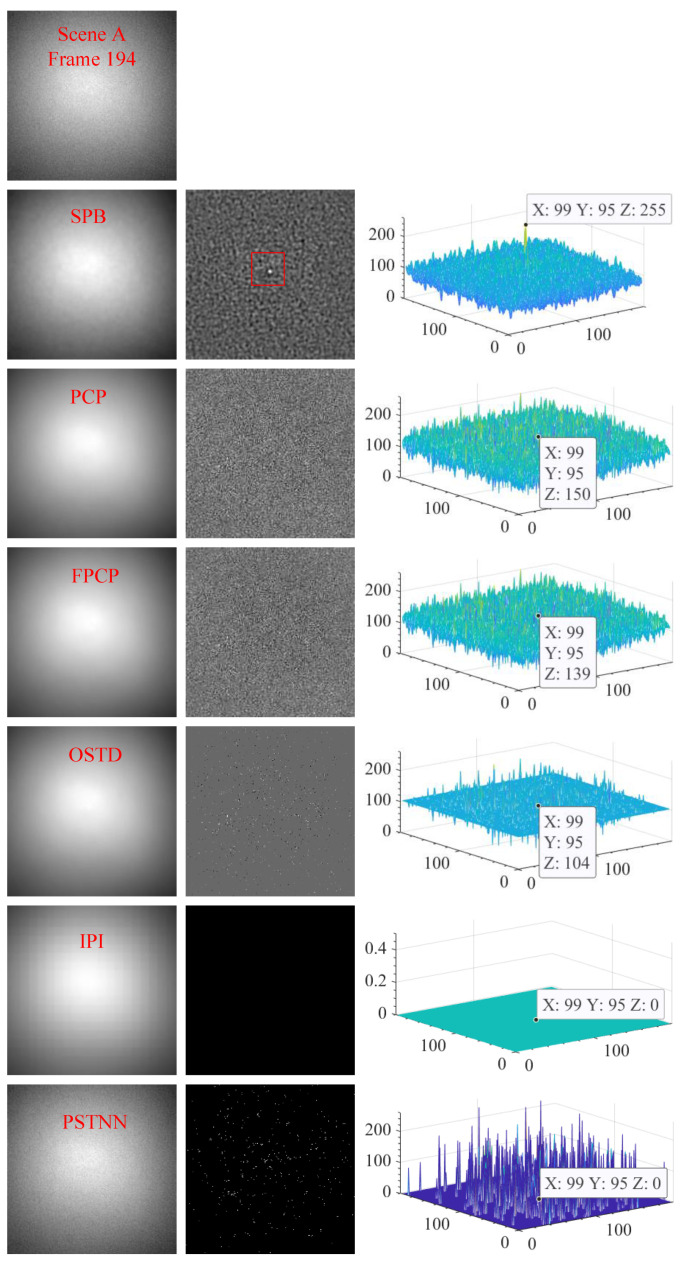
The visual effect of background suppresses of the six algorithms for scene A.

**Figure 13:**
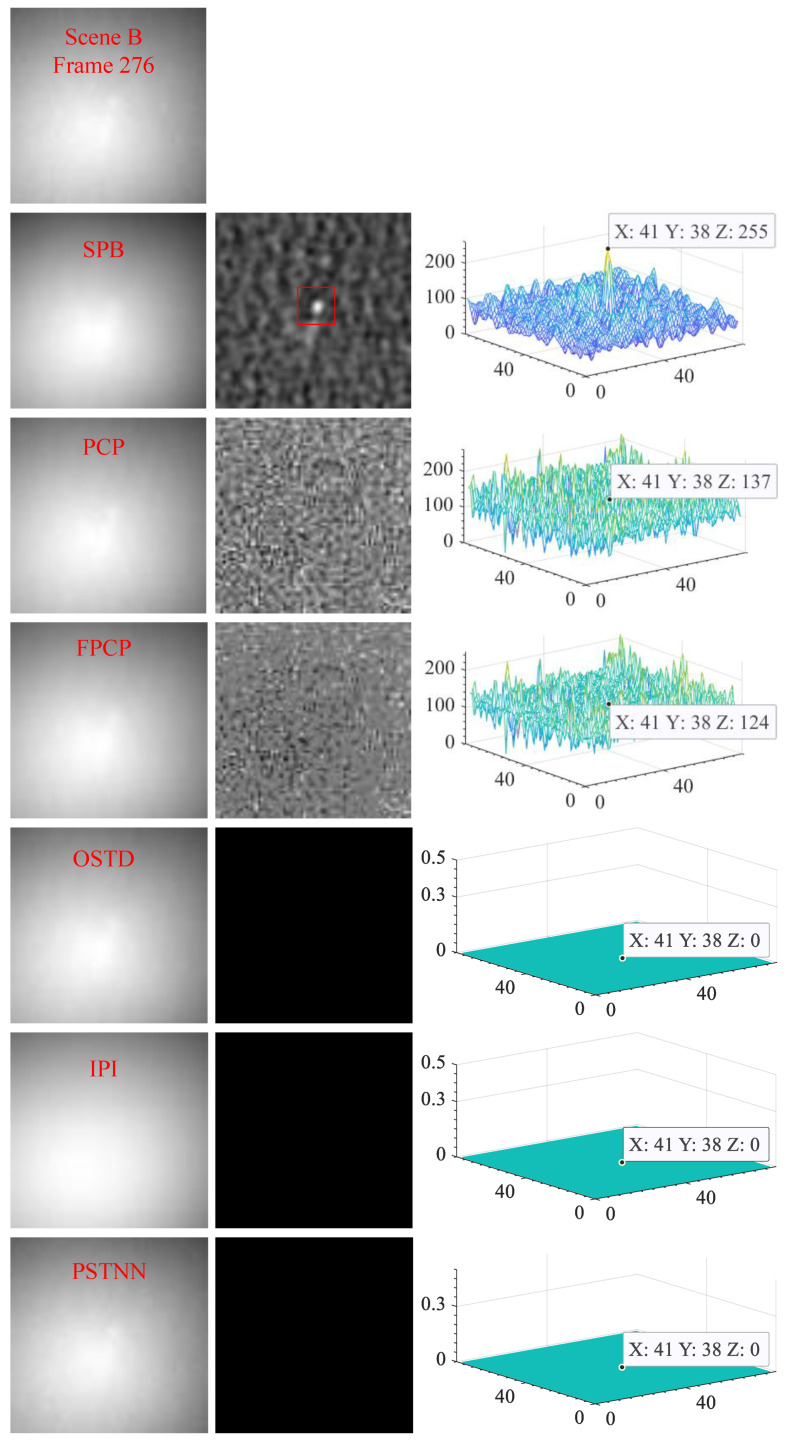
The visual effect of background suppresses of the six algorithms for scene B.

**Figure 14:**
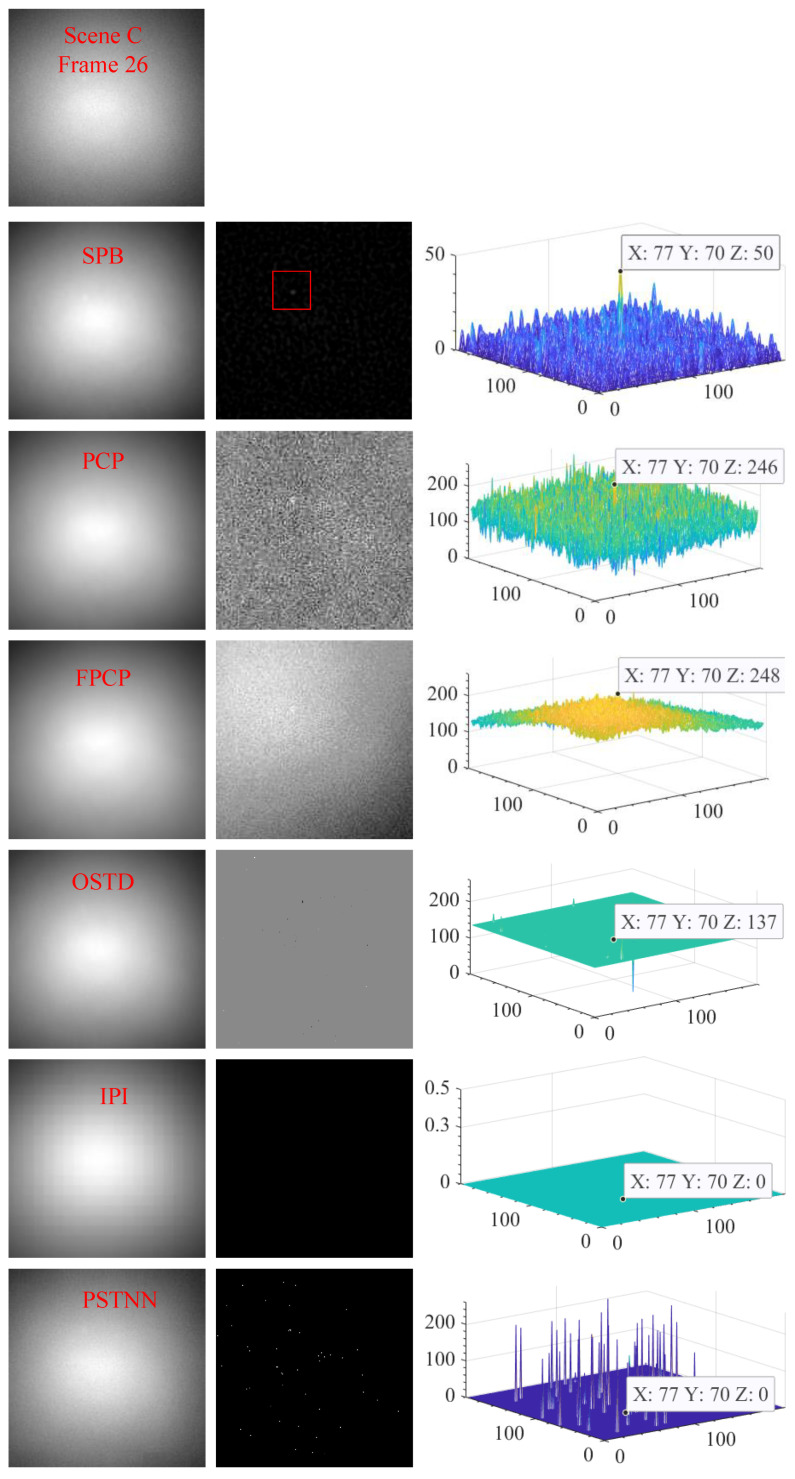
The visual effect of background suppresses of the six algorithms for scene C.

Figure 12, Figure 13 and Figure 14 show the visual comparison of the background suppression effect between the SPB algorithm and the PCP, FPCP, OSTD, IPI, and PSTNN algorithms. These three sets of pictures correspond to scenes A, B, and C respectively, and one frame image is randomly selected from each scene. The first row of each set is the original video frame image. In each set, from the second row to the seventh row, the background image is on the left, the difference image is in the middle, and the three-dimensional energy distribution image is on the right. For the strong sky scene, the experimental results demonstrate that the six algorithms all have the ability to suppress the background, but the separation effect of sparse components and the target enhancement ability is very different. The difference images of SPB, PCP, and FPCP all contain sparse components, but only the difference image of SPB could effectively obtain the target that is significantly enhanced. OSTD, IPI, and PSTNN could not effectively separate the sparse components, that is, there is no effective sparse component in their difference images.

### 4.3. Quantitative Evaluation

In addition to the SPB algorithm proposed in this paper, which can effectively separate dim-small targets from the strong vignetting background, the other five algorithms are unable to effectively separate targets from the background, that is, the difference images of these five algorithms cannot contain effective target information. Therefore, only a quantitative analysis of the SPB algorithm was carried out in this section.

The input SNR (${\mathrm{SNR}}_{\mathrm{in}}$) directly affects the difficulty of separating the dim-small target from the background. The smaller the ${\mathrm{SNR}}_{\mathrm{in}}$, the more difficult it is to separate the target from the background. Therefore, whether a good target enhancement effect can be achieved under different ${\mathrm{SNR}}_{\mathrm{in}}$ can be used to measure the operating range (OR) of an algorithm. The lower the effective ${\mathrm{SNR}}_{\mathrm{in}}$, the wider the OR of the algorithm. As shown in Table 3 and Table 4 and Figure 8, when ${\mathrm{SNR}}_{\mathrm{in}}\geq -12.0820\mathrm{dB}$, the SPB algorithm can effectively separate the target from the background. Thus, the operating range of the SPB is OR≥−12.0820 dB. The stronger the enhancement ability of an algorithm to the target, the higher the value of SNRG. In Table 4, the mean SNRG values of scenes A, B, and C are 3.0103 dB, 5.5784 dB, and 6.6343 dB, respectively, indicating that the SPB algorithm can effectively enhance the target in the working range of OR≥−12.0820 dB. BSF measures the ability of an algorithm to suppress the background. The better the ability of an algorithm to suppress the background, the more evenly the residual noise distribution of the difference image, the smaller the variance of the difference image, and the larger the BSF value. In Table 4, the mean BSF values of the SPB algorithm for scene A, B, and C are 2.8061, 1.7039, and 13.8459, respectively, indicating that the algorithm performs effectively to suppress the background.

## 5. Conclusions

SPB is a novel single-frame background modeling algorithm based on the background space basis function. The algorithm achieves good background suppression and target enhancement performance, which is mainly reflected in the following aspects. Firstly, SPB is robust to targets with different SNR, velocity, and trajectory. Experiments demonstrate that the SPB could effectively separate the dim-small target from the background under extremely low SNR (SNR<1.5dB), moving speed up to 2 pixels/frame, and complex trajectory. Secondly, the residual noise could be well whitened, whose spectrum is evenly distributed. Finally, the target has been significantly enhanced. When the averages of the input SNR of Scene A, B, and C were 3.0237 dB, − 0.2066 dB, and 2.8393 dB, respectively, the SNR gain mean values obtained by SPB are 3.0103 dB, 5.5784 dB, and 6.6343 dB, respectively. Meanwhile, the corresponding mean values of background suppress factor (BSF) are 2.8061, 1.7039, and 13.8459, respectively.

At present, SPB only investigates matrix features from the perspective of the spatial domain, while future works can be carried out from the perspective of the time domain and spatial domain fusion. The main shortcoming of the SPB algorithm is a bit complex and time-consuming due to the fact that to estimate the background, it needs to traverse the entire video frame image pixel by pixel.

## Figures and Tables

**Table 1:** The data of the sparse factors and their corresponding regional scale.

**Scales**	247	201	101	91	81	71	61	51	41	31
**SF**	0.4481	0.4788	0.4919	0.4903	0.4893	0.4888	0.4888	0.4893	0.4902	0.4921
**Scales**	21	19	17	15	13	11	9	7	5	
**SF**	0.4947	0.4952	0.4956	0.4959	0.4962	0.4965	0.4965	0.4967	0.4965	

**Table 2:** The parameters of 6 algorithms.

Algorithms	Parameters
SPB	Local region size: 3a×3a, local block size: a×a, $\lambda =1/\sqrt{\max(\mathrm{size}(D))}$ μ=0.25/mean(D)$,\;\varphi =10^{-7}$. Here, *a* is the target scale.
PCP	$\lambda =1/\sqrt{\max(\mathrm{size}(D))}$, μ=0.25/mean(D)$,\;\varphi =10^{-7}$
FPCP	$\lambda =1/\sqrt{\max(\mathrm{size}(D))}$, lambdaFactor = 1.0; initial rank is r = 1, rankThreshold = 0.01
OSTD	$\lambda_{1}=1/\sqrt{\max(\mathrm{size}(D))}$, $\lambda_{2}=10\lambda_{1}$
IPI	Patch size: 50×50$,\;\mathrm{sliding}\;\mathrm{step}:10,\;\lambda =1/\sqrt{\min(\mathrm{size}(D))}$, $\varphi =10^{-7}$
PSTNN	Patch size: 40×40, sliding step:40, $\lambda =0.6/\sqrt{\max(\mathrm{size}(D))}$$,\;\varphi =10^{-7}$

**Table 3:** The energy, velocity, and trajectory characteristics of the target.

Scenes	Frames	Sizes	${\mathrm{SNR}}_{\mathrm{in}}$ (dB)			Velocities (Pixels/Frame)			Trajectories
			Min	Max	Mean	Min	Max	Mean	
A	298	250 × 250	−0.2829	4.5780	3.0237	0	1	0.0369	Close to an inverted V.
B	487	120 × 120	−1.7309	1.0886	−0.2066	0	1	0.0021	Swinging near the original position
C	178	250 × 250	−12.0820	11.1423	2.8393	0	2	0.3599	Reciprocating moves with a complex trajectory

**Table 4:** The SNRG and BSF values of SPB algorithm.

Scenes		BSF			SNRG	
	Min	Max	Mean	Min	Max	Mean
A	1.9916	4.3897	2.8061	1.8530	4.7622	3.0103
B	1.2775	2.1021	1.7039	4.1573	6.8882	5.5784
C	8.6084	37.7113	13.8459	2.0991	25.5206	6.6343

## Data Availability

The data used to support the findings of this study are included within the article.
